# High-grade Burkitt Lymphoma Presenting as a Buttock Mass and Foot Drop

**DOI:** 10.7759/cureus.3368

**Published:** 2018-09-26

**Authors:** Komal Ejaz, Qalb A Khan, Muhammad A Raza, Roy Sonia Ahmed, Abdul Aleem

**Affiliations:** 1 Intrenal Medicine, Sheikh Zayed Hospital, Lahore, PAK; 2 Internal Medicine, Wellspan Good Samaritan Hospital, Lebanon, USA; 3 Internal Medicine, Jinnah Hospital Lahore/Allama Iqbal Medical College, Lahore, PAK; 4 Internal Medicine, Quaid-E-Azam Medical College, Bahawalpur, PAK; 5 Internal Medicine, St. Mary Mercy Hospital, Livonia, USA

**Keywords:** burkitt lymphoma, non-hodgkin lymphoma

## Abstract

Burkitt lymphoma (BL), a highly aggressive B-cell non-Hodgkin lymphoma (NHL), usually presents in children and young adults with large extranodal masses involving jaw bones, gastrointestinal tract, and central nervous system. The three main subtypes of BL are endemic, sporadic, and immunodeficiency variant. Extranodal involvement is common in each variant of BL, although muscle tissue involvement is distinctly rare. Mode of spread may be hematogenous or via direct extension of the primary tumor. In this report, we present a case of a 41-year-old male who presented with a palpable mass in the buttock leading to foot drop as the initial manifestation of BL. An exhaustive review of the literature failed to discover any previous reports of BL occurring in this location.

## Introduction

Lymphoma is defined as a neoplastic proliferation of lymphoid cells at different stages of differentiation and affects lymph nodes, but may infiltrate into the bone marrow, spleen, and the thymus. Lymphoma is ranked as the seventh most common malignancy in both males and females combined. Extranodal involvement is recognized as 25%-40% of cases of lymphomas and almost any organ can be involved. Extranodal lymphoma, by definition, involves sites other than lymph nodes, spleen, thymus, and the pharyngeal lymphatic ring [1]. Burkitt lymphoma (BL) is a type of non-Hodgkin lymphoma (NHL) that frequently involves extranodal sites. To the best of our knowledge, this is the first case report of BL presenting as a buttock mass and foot drop as the initial manifestation.

## Case presentation

 A 41-year-old Ukrainian male with a past medical history of acquired immunodeficiency syndrome (AIDS) and syphilis presented to the emergency department with a complaint of sudden-onset left buttock pain for one day. The pain was described as severe, progressive, continuous, radiating to the left foot, associated with numbness and burning sensation of the left foot and foot drop. The patient also reported a palpable mass in the left buttock. The patient was diagnosed with human immunodeficiency virus (HIV) seven years ago and his most recent cluster of differentiation (CD)4 count was 20; he was not receiving treatment for AIDS. He was also a diagnosed case of latent syphilis for 12 years. His past surgical and family history was non-contributory. He denied smoking as well as alcohol and illicit drug abuse. The patient was sexually active with one male partner. The patient denied any fever, back pain, urinary/bowel habit changes, swelling in any other part of the body, history of trauma, sexual dysfunction, and appetite/weight changes.

Pertinent findings during the physical examination of the lower limbs include diminished deep tendon reflexes (DTRs) of the left lower extremity, with a static response of plantar along with left foot drop. Sensation to touch was diminished on the left side in the L5, S2, and S3 dermatomal areas and was completely absent below the left ankle. The motor and sensory examination of the right lower extremity was completely normal. Local exam of the left buttock showed a firm, fluctuant, freely mobile mass measuring 4 x 4 cm with no overlying skin changes.

**Figure 1:**
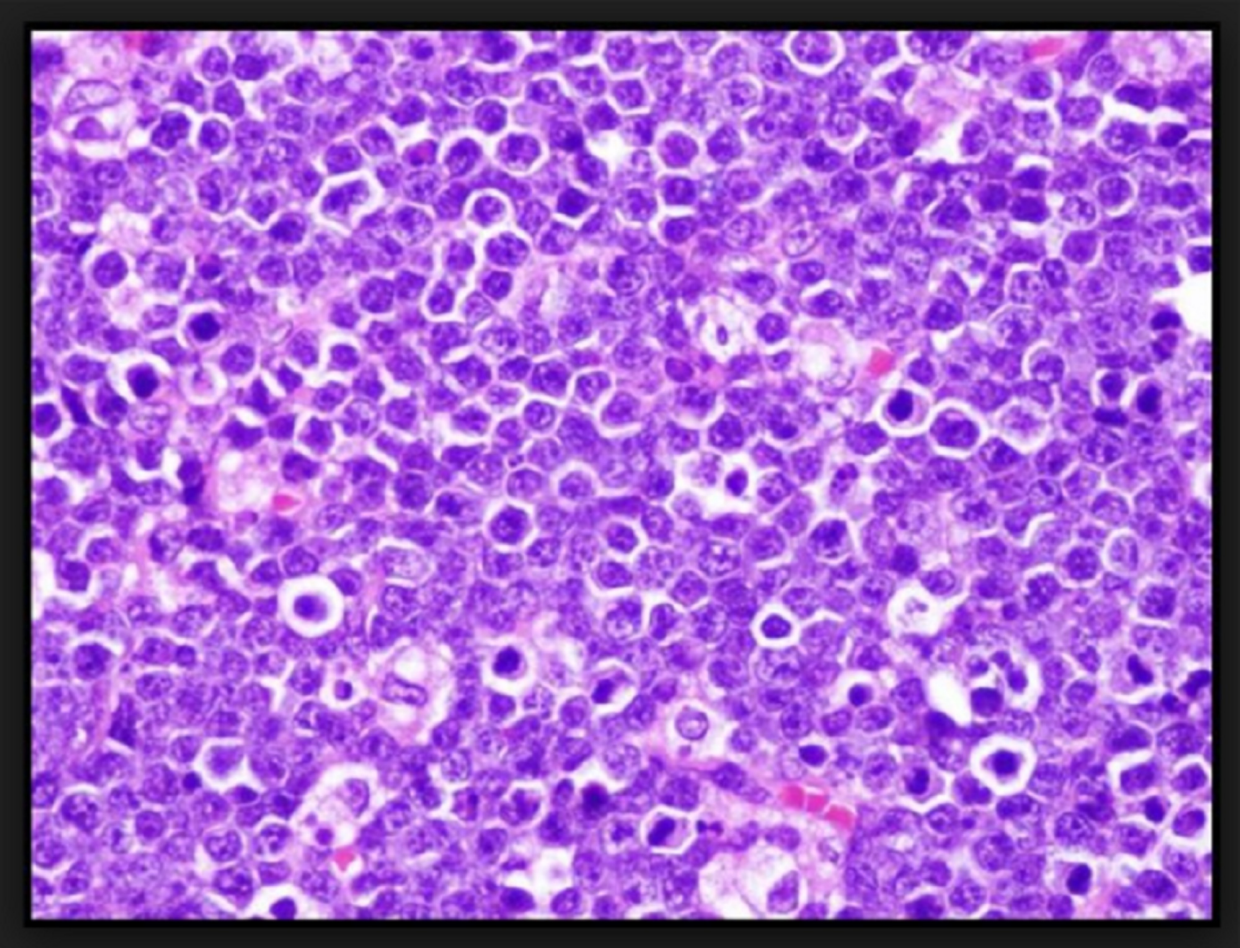
Medium-sized lymphocytes with scant cytoplasm, and presence of numerous tangible-body macrophages

The baseline investigations were unremarkable except hemoglobin (Hb): 9 g/dl, blood urea nitrogen (BUN): 18 mg/dl, and serum creatinine (Cr): 1.25 mg/dl. A left hip X-ray revealed a soft tissue density. Computed tomography (CT) scan of the pelvis confirmed a solid mass. A biopsy of the left buttock mass was performed which showed high-grade BL, a type of NHL (Figure 1). The patient was referred to the oncology department and was started on intensive high dose chemotherapy along with highly active antiretroviral therapy (HAART).

## Discussion

NHL consists of a group of malignant lymphoproliferative disorders arising primarily in the lymph nodes with heterogeneous clinical and histological characteristics. At least 25% of NHLs have been found to originate from tissues other than lymph nodes and sometimes even from sites that are completely devoid of lymphoid tissue [2]. These are referred to as primary extranodal lymphomas (pENLs). Owing to their varied clinical presentations, molecular alterations, and morphological mimicry, pENL is a frequent diagnostic challenge to pathologists and clinicians. Over the last two decades, a rapid increase has been noted in the incidence of lymphomas arising in extranodal sites, especially in the gastrointestinal tract (GIT), the central nervous system, and the skin. This increment may be attributed to chronic infections, immunosuppressive disorders such as AIDS, environmental factors, autoimmune disorders, and immunosuppressive treatments [3].

BL, a highly aggressive mature B-cell NHL has been classified by the World Health Organization (WHO) classification of lymphoid neoplasms as endemic, sporadic, and immunodeficiency-associated variants. Despite the presence of a considerable overlap, each variant displays unique genetic and clinical features. The endemic form is most commonly observed in children aged four to seven years in equatorial Africa, commonly involving the jaw and kidneys, although ileal, cecal, breast, and ovarian involvement have also been reported. This variant has been termed as endemic owing to its remarkably high incidence in equatorial Africa (50 times higher than in the United States) and the identical geographic distribution of this variant and endemic malaria. In contrast, the sporadic variant has not been associated with any specific geographic or climatic distribution, mostly presenting as an abdominal tumor. This clinical variant accounts for 1%-2% of all adult lymphomas in the United States and Western Europe [4].

The immunodeficiency variant of BL, as seen in our patient, is commonly observed in the setting of HIV infection; however, unlike other HIV-related lymphomas, it has been reported in patients with CD4 counts above 200 cells/μL and those with no opportunistic infections [5]. Adult patients inflicted by sporadic or immunodeficiency-associated BL variants typically present with the extranodal disease, most frequently involving the abdomen. The advent and increased use of potent antiretroviral therapy (ART) have failed to decrease the incidence of immunodeficiency variant of BL despite a considerable decline in the incidence of other HIV-associated lymphomas.

The hallmark of BL is the overexpression of c-myc, most commonly the result of translocation (8;14), which causes the juxtaposition of the myc gene from chromosome 8 with the immunoglobulin (Ig) H region on chromosome 14 [6]. The aberrantly high myc expression enhances B-cell proliferation irrespective of the presence of Epstein-Barr virus (EBV) infection [7]. According to the French-American-British classification, BL tumor cells are characterized by the expression of B-cell-specific surface markers, including the cluster of differentiation (CD)19 and CD20, and IgM, and Burkitt-like morphology [8].

Numerous staging systems have been formulated for BL due to increasing frequency of extranodal disease. Ann Arbor system is commonly referenced in adult trials, however, some researchers find this system inadequate due to its inability to fully describe the extent of extranodal involvement. Therefore, some trials prefer St Jude or Murphy staging schema. Notably, this staging system recognizes Burkitt leukemia as a separate entity, unlike the current WHO classification [9]. During the last few decades, CT and MRI scans have been used as the primary imaging modalities for the staging and restaging of lymphoma [10]; however, they may fail to recognize BL in the early stages. Recently, a number of studies have pointed out 18-fluorodeoxyglucose positron-emission tomography/CT (FDG-PET/CT) may have greater sensitivity when compared to anatomic imaging modalities [11]. At the time of diagnosis, patients usually have elevated lactate dehydrogenase (LDH) and uric acid levels. High serum LDH level is associated with advanced-stage disease and poor prognosis in BL [12].

With the use of aggressive chemotherapy, the survival rates of patients with BL have improved particularly for children and adolescents, with a five-year survival rate of 85%-90% [13]. In contrast, the adverse side effects of aggressive chemotherapy such as drug toxicity and severe infections are not well tolerated by most of the adults and elderly patients. Therefore, no definite chemotherapeutic regimens have been established for high-grade BL [14]. However, the introduction of anti-CD20 monoclonal antibody, rituximab, and/or allogeneic hematopoietic stem transplantation has improved outcomes for adult patients, especially those at high-risk [15]. HIV positive patients can be treated with intensive therapy but may additionally require close monitoring, transfusion support, and antibiotic therapy. HAART may improve outcome and allows patients to better tolerate full-dose chemotherapy [16].

## Conclusions

The present case, to the best of our knowledge, is the first to document buttock mass with foot drop as the initial manifestation of BL. BL involving such atypical sites can pose a diagnostic challenge to clinicians. Immunodeficiency variant of BL should be kept in mind in an HIV-positive patient presenting with a suspicious mass in an unusual site (such as the buttock as in this case), and a thorough evaluation including imaging and biopsy should be carried out.
